# Glycated Albumin and Glycated Albumin/HbA1c Predict the Progression of Coronavirus Disease 2019 from Mild to Severe Disease in Korean Patients with Type 2 Diabetes

**DOI:** 10.3390/jcm11092327

**Published:** 2022-04-21

**Authors:** Jeongseon Yoo, Youngah Choi, Shin Ae Park, Ji Yeon Seo, Chul Woo Ahn, Jaehyun Han

**Affiliations:** 1 Department of Internal Medicine, Ilsan Cha Hospital, Goyang-si 10414, Korea; kidturtle@chamc.co.kr; 2 Graduate School, Yonsei University College of Medicine, Seoul 03722, Korea; 3 Department of Internal Medicine, Seobuk Hospital, Seoul Metropolitan Government, Seoul 03433, Korea; youngah12@seoul.go.kr; 4 Department of Family Medicine, Seobuk Hospital, Seoul Metropolitan Government, Seoul 03433, Korea; 24773@seoul.go.kr (S.A.P.); pobbii4@seoul.go.kr (J.Y.S.); 5 Department of Internal Medicine, Gangnam Severance Hospital, Yonsei University College of Medicine, Seoul 06273, Korea; 6 School of Business, Kwangwoon University, Seoul 01897, Korea

**Keywords:** COVID-19, SARS-CoV-2, type 2 diabetes mellitus, glycated hemoglobin a, glycated albumin to glycated hemoglobin ratio, glycosylated serum albumin, South Korea

## Abstract

Hyperglycemia is among the main risk factors for severe COVID-19. We evaluated the association of glycated albumin (GA) and GA/HbA1c ratio with progression of COVID-19 from mild to severe disease in patients with type 2 diabetes mellitus (T2DM). Our retrospective study included 129 patients aged over 18 years with COVID-19 and T2DM who did not have any need of oxygen supplement. Of these, 59 patients whose COVID-19 was aggravated and required oxygen supplementation eventually were classified as having severe disease. Clinical and laboratory data were compared between mild and severe cases. The median of GA (18.4% vs. 20.95%, *p* = 0.0013) and GA/HbA1c (2.55 vs. 2.68, *p* = 0.0145) were higher in severe disease than in mild disease and positively correlated with C-reactive protein (Kendal Tau coefficient 0.200 and 0.126, respectively; all *p* < 0.05). Multiple logistic regression analysis showed that GA (odds ratio (OR), 1.151; 95% confidence interval (CI), 1.024–1.294) and GA/HbA1c (OR, 8.330; 95% CI, 1.786–38.842) increased the risk of severe disease. Patients with GA 20% or higher were 4.03 times more likely to progress from mild to severe disease. GA and GA/HbA1c ratio predicted progression of COVID-19 from mild to severe disease in patients with T2DM.

## 1. Introduction

Coronavirus disease 2019 (COVID-19) has spread globally and has caused widespread catastrophic damage [1]. South Korea is no exception [2]. However, there is a wide spectrum of manifestations, ranging from asymptomatic to death, due to acute respiratory distress syndrome. Therefore, the government stratified patient care. They classified and assigned patients with COVID-19 to stay at home or one of several facilities, such as COVID-19 community treatment center, dedicated COVID-19 hospitals, and respiratory care split hospitals, according to severity of the disease, as determined by an epidemiologic survey [3]. Patients were discharged or transferred according to the patients’ medical conditions and government guidelines [4]. However, there was acute shortage of medical resources at the beginning of each outbreak not only for beds for patients but also for ambulances because these facilities are located in different locations [5]. Therefore, in order to predict the demand for each type of facility and avoid confusion due to misclassification, accurate assessment of the risk at an early stage is of the utmost importance.

Diabetes mellitus (DM) and hyperglycemia per se are known to be the medical conditions that exacerbate COVID-19. DM increases the risk of mortality, intensive care unit (ICU) admission, and severity of disease [6] due to the hyperglycemia-induced dysregulated immune system [7]. HbA1c is the current gold standard for assessing long-term glycemic control for 90–120 days. Whether high HbA1c levels can predict poor outcomes of COVID-19 is controversial [8,9,10]. Glycated albumin (GA) is the marker that can monitor glycemic control over 2–3 weeks. GA is useful for patients with hemoglobinopathy, renal failure, and pregnancy. Furthermore, GA better reflects postprandial hyperglycemia, nephropathy, retinopathy, arterial stiffening, peripheral vascular calcification, and Alzheimer’s disease than HbA1c. The GA/HbA1c ratio correlates significantly with glucose excursion and insulin secretory function [11,12,13]. However, to the best of our knowledge, no studies have analyzed GA and GA/HbA1c ratio in COVID-19 patients. 

In this study, we evaluated the impact of glucose control status represented by GA and GA/HbA1c ratio on COVID-19 severity.

## 2. Materials and Methods

### 2.1. Patients

During the period from 1 June 2021 to 30 November 2021, 1948 patients with COVID-19 were admitted to Seobuk Hospital located in Seoul of South Korea, which is a dedicated hospital for mild COVID-19. Inclusion criteria were as follows: (1) Koreans aged 18 years or older with (2) SARS-CoV-2 infection laboratory-confirmed by real-time reverse transcriptase-polymerase chain reaction (RT-PCR) assay of nasopharyngeal swab samples and (3) previous or newly diagnosed type 2 diabetes mellitus (T2DM). Exclusion criteria were as follows: (1) pregnancy, (2) users of immunomodulating drugs for any reason (e.g., ulcerative colitis, rheumatoid arthritis), (3) uncompensated liver cirrhosis, (4) recent event of cardio- or cerebro-vascular disease within 6 months, (5) uncontrolled malignancy, (6) active lung disease (e.g., asthma, COPD) other than pneumonia of COVID-19, (7) severe COVID-19 disease that needed O_2_ supplement on first diagnosis, (8) end-stage renal disease with renal replacement therapy, and (9) any disease related to hypoalbuminemia (e.g., nephrotic syndrome, heart failure).

One hundred and thirty-one patients who were younger than 18 years and 1580 patients with normal serum glucose and no previous diagnosis of DM were excluded from the study. One hundred and thirty-eight patients had results of both HbA1c and GA tests taken within 3 days after admission. Among them, nine patients were excluded because of the following reasons: five foreigners, two patients with undiagnosed liver cirrhosis, and two with chronic obstructive pulmonary lung disease. Finally, 129 patients were included in our analyses.

Oxygen supplementation was started if the patient’s SpO_2_ was equal to or below 94% in room air, and FiO_2_ was increased until patient’s SpO_2_ recovered above 95%. We defined this condition requiring any oxygen supplementation as severe disease according to the World Health Organization COVID-19 ordinal scale for clinical improvement [14].

### 2.2. Collection of Medical Data

The patients’ reports from an epidemiologic survey by local government and electronic medical records were reviewed. Before and after admission, registered nurses and physicians rechecked the details and corrected them if needed. Data on age, sex, comorbidities, vital signs, laboratory findings, medical treatment, and clinical outcomes were collected. Anthropometric measurements and blood collection were performed on the first day of admission, and thereafter, the test intervals varied depending on the medical condition. Vital signs, including blood pressure, pulse, body temperature, respiratory rate, and SpO_2_, were measured by the patients themselves if they were capable. Laboratory testing for HbA1c, GA, fasting blood sugar (FBS), triglycerides, and LDL-cholesterol, which had to be done while fasting, were performed within 3 days after admission.

DM was defined as any of the following: (1) previous diagnosis of DM by physician(s), (2) taking prescribed antihyperglycemic agents, or (3) HbA1c ≥ 6.5%. Therefore, not only pre-existing DM but also DM newly diagnosed after admission were included in this study. Dyslipidemia was defined as any of the following: (1) previous diagnosis of dyslipidemia by physician(s) or (2) taking prescribed lipid-lowering drug(s). Hypertension, ischemic heart disease, stroke, and previous malignant disease were recorded according to previous diagnosis by physician(s).

### 2.3. Laboratory Assay and Calculation of Estimated Glomerular Filtration Rate (eGFR)

HbA1c was measured in the laboratory of Seobuk Hospital by immunoturbidimetric assay method, certified by National Glycohemoglobin Standardization Program, using AU HbA1c reagent (Beckman Coulter, Inc., Brea, CA, USA) and AU680 Clinical Chemistry Analyzer (Beckman Coulter, Inc., Brea, CA, USA) as per the manufacturer’s instructions. 

Serum GA level measurement was performed by Seoul Clinical Laboratories (Yongin-si, Gyeonggi-do, Korea) and determined by enzymatic method using JW Reagent-GA (JW Bioscience, Seoul, Korea) and Toshiba TBA c8000 (Toshiba Medical. Systems Ltd., Tokyo, Japan) as per the manufacturer’s instructions. Serum GA levels were calculated as the percentage of GA relative to total albumin. Normal control of serum GA levels for adults is from 11.0 to 16.0%.

We used CKD-EPI Creatinine Equation (2021) [15] to eGFR, recommended by the National Kidney Foundation and the American Society of Nephrology, as below.

eGFRcr = 142 × min (S_cr_/κ, 1)^α^ × max(S_cr_/κ, 1)^−1.200^ × 0.9938^Age^ × 1.012 (if female)

where:

S_cr_ = serum creatinine in mg/dL;
κ = 0.7 (females) or 0.9 (males);
α = −0.241 (female) or −0.302 (male);
min (S_cr_/κ, 1) is the minimum of S_cr_/κ or 1.0;
max (S_cr_/κ, 1) is the maximum of S_cr_/κ or 1.0; and
Age (years).

### 2.4. Statistical Analysis

**Figure 1:**
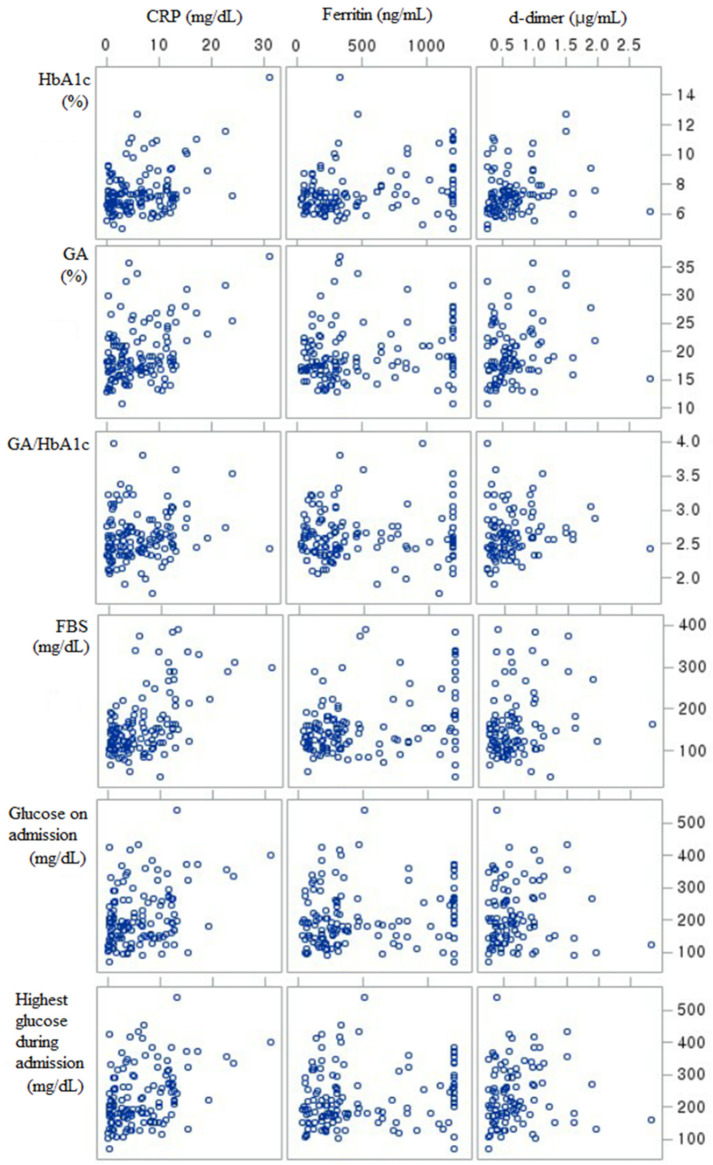
Kendall Tau correlations of glucose control status and inflammatory markers with d-dimer. FBS, fasting blood sugar; GA, glycated albumin; CRP, C-reactive protein.

Normally distributed continuous variables are expressed as mean ± standard deviation and compared using independent *t*-test. Skewed continuous variables are expressed as median and interquartile range (IQR) and compared using the Mann–Whitney U test (Table 1). Kendell’s Tau correlation was used to check the association between continuous variables (Table 2 and Figure 1). Categorical variables were expressed as frequency (n) and percentage (%) and compared using the χ^2^ test, ANOVA (analysis of variance), or Fisher’s exact test. In case of ANOVA, post hoc test was also performed. To determine the effects of glucose control by GA, GA/HbA1c, and HbA1c, univariate and multiple logistic regression analyses were performed (Table 3 and Table 4). For logistic regression, we could not analyze ischemic heart disease and stroke because the number of patients was not enough. Furthermore, there were no patients with ischemic heart disease in the severe disease group. In multiple logistic regression (Table 4), we selected the variables that showed statistical significance in age-adjusted logistic regression and performed adjustment by age, sex, body mass index (BMI), time to admission from onset of COVID-19-related symptoms, systolic blood pressure (SBP) on admission, and completion of vaccine. In cases of worst glucose during admission and fasting blood glucose (FBS), we added dexamethasone use for the adjustment. All statistical analyses were performed using SAS (version 9.4; SAS Institute, Cary, NC, USA), and statistically significant differences were reported at *p* < 0.05.

### 2.5. Ethical Approval Statement

This study protocol was reviewed and approved by the Institutional Review Board of Seobuk Hospital (approval No. 116272-211129-HR-008-01). Informed consent was waived because of the retrospective nature of the study design.

## 3. Results

This section may be divided by subheadings. It should provide a concise and precise description of the experimental results, their interpretation, as well as the experimental conclusions that can be drawn.

### 3.1. Baseline Characteristics of Study Subjects

More than half of our study subjects were older than 63 years. The median age of patients with severe disease was 10 years younger than that of patients with mild disease. Our study population was male-dominated. Sex and smoking status did not differ between the two groups.

The patients with BMI over 25 kg/m^2^. BMI was higher in the patients with severe disease. However, there were no significant differences of obesity defined with BMI over 30 kg/m^2^ and sex between two groups (Table 1).

Twenty-one patients (16.3%) were newly diagnosed with DM after admission for COVID-19. The proportion of undiagnosed DM was 7.4% higher in patients with severe disease than those with mild disease, but there was no statistical significance (*p* = 0.2515). Over 50% of the patients had been previously diagnosed with hypertension or dyslipidemia. There was no significant difference of lipid profile between two groups. More than 80% of patients had normal kidney function with eGFR > 60 mL/min/1.73 m^2^. About one-third of the patients completed two doses of vaccination (Table 1). Because many patients did not remember the exact date of last vaccination, and it had been less than two weeks since the second vaccination in many cases, all cases with completion of both vaccinations did not mean breakthrough infection. Nevertheless, patients who received two doses of vaccination were less than half as likely to progress to severe disease.

### 3.2. Differences between Patients with Mild and Severe Disease

Although our patients were all classified as having mild disease at the time of COVID-19 confirmed by RT-PCR, the vital signs and laboratory findings of many patients with mild disease were significantly worse than those in patients with severe disease on the first day of admission. Body temperature was slightly higher in patients with severe disease even though many of the patients had taken antipyretics before admission. SpO_2_ in patients with severe disease was slightly lower than that in patients with mild disease, and SpO_2_ was sometimes measured during O_2_ supplementation in cases where patients had developed hypoxia before admission.

The DM duration of patients with severe disease was longer than those with mild disease. The proportion of patients with newly diagnosed T2DM right after admission was higher in patients with severe disease than in those with mild disease. However, these were not statistically significant.

Serum creatinine (Cr) and eGFR showed no differences between the two groups on admission and even in the worst condition during admission group. Systolic blood pressure on admission was lower in patients with severe disease than in those with mild disease (Table 1).

Albumin was slightly lower in the patients with severe COVID-19. However, it did not affect to our GA level because it was measured by enzymatic methods after removing glycated amino acid and reported as a percentage of total albumin.

### 3.3. Glucose Control and the Status of COVID-19

HbA1c, GA, GA/HbA1c, and FBS were significantly higher in patients with severe disease. These statistical significances were maintained even when analyzed as categorical variables by the χ^2^ test regardless of cutoff points except GA/HbA1c (Table 1).

HbA1c and GA were significantly correlated with worst CRP, worst ferritin, and worst d-dimer. GA/HbA1c was correlated only with worst CRP and not with worst ferritin, and worst d-dimer (Table 2 and Figure 1).

High HbA1c was an independent risk factor for the need for O_2_ supplementation in COVID-19 patients in unadjusted logistic regression. Patients whose HbA1c was 6.5% or higher were 2.55 times more likely to need O_2_ supplementation (Table 3). However, statistical significance was lost after adjustment by age, sex, BMI, time to admission from onset of COVID-19-related symptoms, systolic blood pressor on admission, completion of vaccination, and whether it was analyzed as a continuous variable or categorical variable. Elevated GA and GA/HbA1c consistently increased the risk of severe disease even after adjustment for confounders. However, we found that GA over 26% and GA/HbA1c over 2.7 as categorical variables did not predict the progression of COVID-19 (Table 4). 

## 4. Discussion

We found that GA and GA/HbA1c ratio predicted the risk of COVID-19 progression from mild to severe disease. To our knowledge, there are no published data on the relationship between COVID-19 severity and GA and GA/HbA1c ratio.

This is the first clinical evidence that short-term glycemic control and sugar fluctuations could have a significant impact on the human immune system as related to COVID-19 infection. GA reflects the status of sugar control for 2–3 weeks, which covers the period before and after contact with SARS-CoV-2. Because the time taken from onset of symptoms to hospitalization was just a few days (Table 1) in our patients, we believe that GA and GA/HbA1c can reflect the glucose control status during the incubation period of COVID-19. Experimental data on the relationship between GA, GA/HbA1c and respiratory infection are scarce. Recently, Aitken et al., demonstrated that human GA triggers an inflammatory response by increasing IL-8 secretion and ciliary beat frequency in human airway epithelial cultures [16].

Elevated HbA1c was an independent risk factor for the need for O_2_ supplementation in COVID-19 patients in unadjusted logistic regression (Table 3). However, HbA1c lost its statistical significance after adjustment with age, sex, BMI, time to admission from onset of COVID-19-related symptoms, and SBP on admission (Table 4). We proposed that even short-term changes in glucose control status could alter the effect of long-term glucose control on the human immune response to SARS-CoV-2. Otherwise, it could be merely because glucose level of our patients was generally well-maintained and therefore did not show significant differences. Only 32 (25%) among 129 patients had HbA1c levels ≥ 8% (Table 1).

The proportion of pre-existing DM per se was not significantly different between patients with mild and severe diseases (87.1% vs. 79.7%, *p* = 0.2515). This finding is consistent with previous studies on infectious diseases [17], including COVID-19 [18].

High glucose level was strongly correlated with COVID-19 severity (Table 3 and Figure 1) and a significant risk factor in our (Table 4) and previous reports [19,20]. However, we considered that glucose levels on and during admission could be a result rather than a cause of COVID-19 aggravation. In fact, all types of glucose levels were not significant statistically in the adjusted logistic regression (Table 4).

As per our results, the lower the systolic blood pressure, the greater the need for O_2_ supplementation, which is contrary to previous reports [21,22]. In our clinical experience, low systolic blood pressure in COVID-19 patients on admission was usually due to dehydration. During COVID-19 outbreak, especially in the fourth wave that began in July 2021, some patients had to endure sustained high fever and poor oral intake for several days during self-quarantine because of the shortage of hospital beds. Normalization of blood pressure usually occurred after intravenous hydration, and there was no significant difference of blood pressure in discharge (Table 1). Therefore, relatively higher systolic blood pressure in mild disease should not be interpretated that uncontrolled hypertension has a protective effect on the progression of COVID-19.

Younger patients had higher odds of requiring O_2_ supplementation than elderly patients in our data. This was because young patients who entered the community treatment center in the first place were transferred to our hospital after aggravation of pneumonia due to COVID-19.

Obesity is one of the major risk factors of severe COVID-19 [23,24]. However, BMI over 30 kg/m^2^ did not have significant impact on COVID-19 aggravation in our study (Table 3). It might be because the number of obese patients was not high enough in our study population, or it could be due to racial differences. In a previous study for elderly Korean patients, underweight with BMI under 18.5 kg/m^2^ was associated with an increased risk of death in COVID-19 patients [25]. 

There are some limitations to our study. First, we could not check the rate of ICU admission, artificial ventilation, and death. The patients whose condition had been aggravated and needed O_2_ supplementation were transferred to other hospitals with an ICU because Seobuk hospital does not have an ICU facility. Therefore, most data on severe disease were censored. Second, we did not have data of respiratory rate and arterial blood gas analysis. Because of the explosive increase in COVID-19 cases and a shortage of medical staff, vital signs with SpO_2_ were checked by patients if possible. Respiratory rate was checked by nurses only in case of severe dyspnea despite O_2_ supplement. Therefore, we excluded respiratory rate for analyses. Third, fructosamine and 1.5-Anhydro-D-Glucitol were not performed because they are subsidized by Korean national health insurance only in case of hemoglobinopathy. Fourth, the previous medical information of the patients was confirmed only through interviews. In most cases, family members of COVID-19 patients had been in self-quarantine or were themselves hospitalized due to COVID-19, and they could not deliver official medical records. However, not only interviewers but also registered nurses and physicians reconfirmed the data by communicating with patients and their families more than twice.

Our study demonstrated that GA and GA/HbA1c ratio can predict the progression of COVID-19 from mild to severe disease. Therefore, they can help identify patients with T2DM who could develop severe COVID-19 eventually. Furthermore, proper support for patients with T2DM during self-quarantine to maintain appropriate sugar control is crucial to prevent the progression of COVID-19 to severe disease.

## 5. Conclusions

GA and GA/HbA1c ratio can predict the progression of COVID-19 from mild to severe disease in Korean adult patients with T2DM.6.

## Figures and Tables

**Table 1:** Characteristics of the study subjects.

	Total	Mild Disease (n = 70)	Severe Disease (n = 59)	*p*-Value
Age (years)	63 (53, 70)	68 (57, 74)	58 (50, 65)	0.0011
Sex (male%)	79 (61.2%)	45 (64.3%)	34 (57.6%)	0.4395
Time to admission from onset of COVID-19-related symptoms (days)	4 (2, 6)	3 (2, 4)	5 (2, 7)	0.0624
Time to developed hypoxia from onset of COVID-19-related symptoms (days)			7.0 (5.0, 8.0)	
Smoking status				0.1939
Non-smokers	100 (77.5%)	50 (71.4%)	50 (84.8%)	
Ex-smokers	7 (5.4%)	5 (7.1%)	2 (3.4%)	
Current smokers	22 (17.1%)	15 (21.4%)	7 (11.9%)	
Past history				
Diabetes mellitus	108 (83.7%)	61 (87.1%)	47 (79.7%)	0.2515
DM duration (years)	4.0 (1.0, 10.0)	5.0 (1.0, 13.0)	3.0 (0.5, 10.0)	0.0850
Newly diagnosed DM	20 (15.5%)	10 (14.3%)	10 (16.95%)	0.6771
Hypertension	75 (58.1%)	42 (60.0%)	33 (55.9%)	0.6408
Dyslipidemia	67 (51.9%)	39 (55.7%)	28 (47.5%)	0.3498
Ischemic heart disease	9 (7.0%)	9 (12.9%)	0 (0%)	0.0038 *
Stroke	7 (5.4%)	5 (7.1%)	2 (3.4%)	0.4525 *
Cancer survivor	10 (7.8%)	12 (17.1%)	2 (3.4%)	0.0124
BMI (kg/m^2^)	25.5 (24.0, 28.5)	24.9 (23.5, 26.7)	26.2 (24.2, 30.2)	0.0145
BMI ≥ 25 kg/m^2^	74 (57.4%)	34 (48.6%)	40 (67.8%)	0.0278
BMI ≥ 30 kg/m^2^	24 (18.8%)	9 (12.9%)	15 (25.9%)	0.0677
On admission				
SpO_2_ (%)	97 (96, 97)	97 (96, 98)	96 (94, 97)	<0.0001
Body temperature (°C)	37.3 (36.9, 37.7)	37.2 (36.8, 37.5)	37.6 (37.0, 38.3)	0.0005
SBP (mmHg)	137.6 ± 19.8	141.9 ± 19.7	132.5 ± 19.0	0.0244
DBP (mmHg)	82.6 ± 11.0	83.9 ± 11.7	81.0 ± 9.9	0.2370
Glucose (mg/dL)	190.0 (146.0, 262.0)	189 (132, 257)	192 (148, 275)	<0.0001
Albumin (mg/dL)	3.80 ± 0.34	3.92 ± 0.28	3.64 ± 0.35	<0.0001
AST (IU/L)	32 (25, 45)	29 (22, 41)	34 (28, 54)	0.0197
ALT (IU/L)	28 (30, 59)	27 (18, 44)	31 (21, 41)	0.5466
Creatinine (mg/dL)	0.96 (0.84,1.12)	0.96 (0.87, 1.12)	0.97 (0.81, 1.12)	0.9313
eGFR (mL/min/1.73 m^2^)	76.05 ± 17.48	75.61 ± 17.27	76.63 ± 17.86	0.5859
Ferritin (ng/mL)	286.6 (162.5, 722.4)	212.2 (101.9, 382.5)	334.1 (221.6, 1017.1)	0.0063
D-dimer (μg/mL)	0.54 (0.38, 0.81)	0.47 (0.34, 0.61)	0.62 (0.43, 0.97)	0.0055
TC (mg/dL)	142 (123, 164)	142.6 ± 30.8	148.2 ± 33.5	0.2815
TG (mg/dL)	127 (106, 165)	128 (115, 151)	124.0 (92.5, 170.5)	0.4181
HDL-C (mg/dL)	41 (37, 48)	43.0 (36.0, 50.0)	40.0 (37.0, 45.5)	0.2011
LDL-C (mg/dL)	75 (63, 92)	78.4 ± 22.4	76.7 ± 27.8	0.7208
Worst laboratory value during admission				
Glucose (mg/dL)	204 (171, 290)	193 (152, 257)	243 (180, 326)	0.0117
Cr (mg/dL)	0.96 (0.84, 1.12)	0.96 (0.87, 1.12)	0.97 (0.81, 1.12)	0.9313
eGFR (mL/min/1.73 m^2^)	73.88 ± 17.68	73.58 ± 17.80	74.24 ± 17.68	0.8326
CRP (mg/dL)	4.54 (1.43, 9.68)	2.23 (0.72, 4.42)	9.80 (5.90, 12.23)	<0.0001
Ferritin (ng/mL)	286.6 (162.5, 722.4)	255.0 (118.6, 425.1)	459.3 (221.6, 1185.7)	0.0040
D-dimer (μg/mL)	0.55 (0.38, 0.81)	0.47 (0.34, 0.61)	0.695 (0.500, 1.025)	0.0005
Sugar control status				
HbA1c (%)	7.1 (6.5, 7.9)	6.95 (6.40, 7.50)	7.30 (6.80, 8.30)	0.0344
HbA1c ≥ 6.5%	101 (78.3%)	50 (71.4%)	51 (86.4%)	0.0364
HbA1c ≥ 7%	76 (58.9%)	35 (50.0%)	41 (69.5%)	0.0250
Glycated albumin (GA) (%)	18.2 (16.5, 21.9)	18.4 (15.5, 19.9)	20.95 (17.4, 24.4)	0.0013
GA ≥ 20%	44 (34.11%)	17 (24.3%)	27 (45.8%)	0.0104
GA ≥ 26%	16 (12.4%)	5 (7.1%)	11 (18.6%)	0.0473
GA/HbA1c	2.57 (2.39, 2.76)	2.55 (2.32, 2.76)	2.68 (2.46, 2.76)	0.0145
GA/HbA1c ≥ 2.7	40 (31.0%)	21 (30.0%)	19 (32.2%)	0.7875
FBS (mg/dL)	143 (112, 183)	122 (106, 152)	164 (136, 268)	<0.0001
Blood pressure on discharge				
SBP (mmHg)	126.4 ± 17.6	125.5 ± 19.0	127.4 ± 15.8	0.5580
DBP (mmHg)	93.0 ± 9.0	92.4 ± 9.3	93.7 ± 8.6	0.4213
Completion of Vaccination				0.0140
Only 1st dose of vaccination	36 (27.9%)	19 (27.1%)	17 (28.8%)	0.2849
Both doses of vaccination	42 (32.6%)	30 (42.9%)	12 (20.3%)	0.0035
Breakthrough infection	4 (7.4%)	2 (7.4%)	2 (7.4%)	1.0000 *
Treatment of COVID-19				
Regdanvimab	77 (59.7%)	43 (61.4%)	34 (57.6%)	0.6610
Remdesivir	55 (42.3%)	6 (8.57%)	49 (83.1%)	<0.0001
Dexamethasone	63 (48.8%)	11 (15.7%)	52 (88.1%)	<0.0001

Data are presented as mean ± standard deviation (SD) if continuous variables satisfied normality or median (interquartile range) for skewed variables. Categorical variables were expressed as frequencies n (%, percent). * Fisher’s exact P. BMI, body mass index; SpO_2_, saturation of partial pressure oxygen; SBP, systolic blood pressure; DBP, diastolic blood pressure; DM, diabetes mellitus; GA, glycated albumin; FBS, fasting blood sugar; TC, total cholesterol; TG, triglyceride; HDL-C, high-density lipoprotein cholesterol; LDL-C, low-density lipoprotein cholesterol; AST, aspartate transaminase; ALT, alanine aminotransferase; Cr, creatinine; eGFR, estimated glomerular filtration rate; CRP, C-reactive protein; SBP, systolic blood pressure; DBP; diastolic blood pressure.

**Table 2:** Kendall Tau correlations of glucose control status and inflammatory markers with d-dimer.

	HbA1c	GA	GA/HbA1c	FBS	Glucose on Admission	Highest Glucose	Worst CRP	Worst Ferritin	Worst D-Dimer
HbA1c	1								
GA	0.6118 †	1							
GA/HbA1c	0.1280 *	0.5261 †	1						
FBS	0.2593 †	0.3158 †	0.1290 *	1					
Glucose on admission	0.3397 †	0.3920 †	0.1748 *	0.2551 †	1				
Highest glucose	0.3715 †	0.4488 †	0.1922 *	0.3840 †	0.77605 †	1			
Worst CRP	0.1554 *	0.2004 *	0.1257 *	0.2907 †	0.16908 *	0.2323 †	1		
Worst ferritin	0.1627 *	0.1463 *	−0.0065	0.1850 *	0.11939 *	0.1359 *	0.2454 †	1	
Worst d-dimer	0.1530 *	0.1431 *	0.1187	0.1202	0.0659	0.1254	0.2973 †	0.0633	1

* *p*-value < 0.05. † *p*-value < 0.001. FBS, fasting blood sugar; GA, glycated albumin; CRP, C-reactive protein.

**Table 3:** Unadjusted logistic regression.

Variable	OR	95% CI
Age (years)	0.959	(0.930, 0.988)
Male Sex	0.756	(0.391, 1.538)
BMI (kg/m^2^)	1.093	(1.003, 1.191)
BMI ≥ 25 vs. BMI < 25	2.229	(1.085, 4.578)
BMI ≥ 30 vs. BMI < 30	2.311	(0.927, 5.756)
Time to admission from onset of COVID-19-related symptoms (days)	1.162	(0.992, 1.361)
SBP on admission (mmHg)	0.975	(0.956, 0.994)
Albumin on admission (mg/dL)	0.065	(0.018, 0.239)
AST on admission (IU/L)	1.013	(0.997, 1.029)
HbA1c (%)	1.319	(1.026, 1.695)
HbA1c ≥ 6.5% vs. HbA1c < 6.5%	2.550	(1.029, 6.322)
HbA1c ≥ 7.0% vs. HbA1c < 7.0%	2.278	(1.102, 4.706)
GA (%)	1.116	(1.032, 1.207)
GA ≥ 20% vs. GA < 20%	2.630	(1.244, 5.562)
GA ≥ 26% vs. GA < 26%	2.979	(0.971, 9.138)
GA/HbA1c	2.571	(0.947, 6.977)
GA/HbA1c ≥ 2.7 vs. GA/HbA1c < 2.7	1.108	(0.525, 2.342)
Glucose (mg/dL)		
Glucose on admission	1.002	(0.998. 1.006)
Worst glucose during admission	1.006	(1.002, 1.011)
FBS	1.017	(1.009, 1.024)
Completion of Vaccination		
Only 1st dose of vaccination vs. never	0.626	(0.265, 1.480)
Both doses of vaccination vs. never	0.280	(0.117, 0.669)

BMI, body mass index; GA, glycated albumin; SBP, systolic blood pressure; AST, aspartate transaminase; FBS, fasting blood sugar; CI, confidence interval; OR, odds ratio.

**Table 4:** Adjusted logistic regression of sugar control markers.

Variable	OR	95% CI
HbA1c (%)	1.169	(0.865, 1.579)
HbA1c ≥ 6.5% vs. HbA1c < 6.5%	2.171	(0.688, 6.849)
HbA1c ≥ 7.0% vs. HbA1c < 7.0%	1.589	(0.642, 3.932)
GA (%)	1.151	(1.024, 1.294)
GA ≥ 20% vs. GA < 20%	4.030	(1.407, 11.540)
GA ≥ 26% vs. GA < 26%	4.655	(0.848, 25.568)
GA/HbA1c	8.330	(1.786, 38.842)
GA/HbA1c ≥ 2.7 vs. GA/HbA1c < 2.7	2.204	(0.792, 6.134)
Glucose (mg/dL)		
Glucose on admission	1.004	(0.998, 1.010)
Worst glucose during admission *	1.006	(0.988, 1.024)
FBS *	1.003	(0.978, 1.028)

Adjusted by age, sex, body mass index, time to admission from onset of COVID-19-related symptoms, systolic blood pressure on admission, and completion of vaccination. * Adjusted by age, sex, body mass index, time to admission from onset of COVID-19-related symptoms, systolic blood pressure on admission, completion of vaccination, and dexamethasone use. GA, glycated albumin; FBS, fasting blood sugar; CI, confidence interval; OR, odds ratio.

## Data Availability

The data presented in this study are available on request after obtaining additional permission of Seobuk Hospital’s Institutional Review Board. They allow only designated researchers to access to data due to the Korean Act on the Protection of Personal Information and patients’ privacy.
